# Sequence and analysis of the complete mitochondrial genome of the Eurasian tree sparrow *Passer montanus saturatus* in the Republic of Korea (Passeriformes, Passeridae)

**DOI:** 10.1080/23802359.2021.1927876

**Published:** 2021-05-19

**Authors:** Ju-hyun Lee, Se-yeong Kim, Dong-yun Lee, Wan-hee Nam, Ha-cheol Sung

**Affiliations:** a School of Biological Sciences and Biotechnology Graduate School, Chonnam National University, Gwangju, Korea; b Department of Biological Sciences, College of Natural Sciences, Chonnam National University, Gwangju, Korea; c Research Center of Ecomimetics, Chonnam National University, Gwangju, Korea

**Keywords:** Complete mitochondrial genome, Passeridae

## Abstract

Eurasian Tree Sparrow *Passer montanus* is widely distributed passerine bird, and one sub-species *P. m. saturatus* is known to inhabit North-East Asia. In this study, we decode the complete mitochondrial genome of *P. m. saturatus* from the Republic of Korea. Mitogenome was 16,904 bp in length, and the content of A, T, G, and C were 30.0% (5079 bp), 22.5% (3810 bp), 15.5% (2621 bp), and 31.9% (5394 bp), respectively. The circular mitogenome contained 38 genes (13 protein-coding genes, 22 transfer RNAs, and 2 ribosomal RNAs) and a non-coding region. Phylogenetic analysis based on the complete mitogenome sequences indicated genetic distances in the species of Passeriformes, and *P. m. saturatus* in the Republic of Korea is included in a monophyletic group with *P. montanus* in China. This result provide basic information of population genetics of wide-ranging species Eurasian Tree Sparrow.

## Introduction

Eurasian Tree Sparrow, *Passer montanus*, is a small passerine bird (family Passeridae) and is a very wide-ranging species. Its native range is distributed from Western Europe to Central Asia, East Asia, Indo-Chinese countries, and the Philippines, and was introduced to United States (Summer-Smith 1995). This species is reported to extant eleven geographic subspecies, of which three subspecies are distributed in the Northeast Asia: *P. m. montanus* (Linnaeus, 1758; in NE Mongolia, NE China, Russia), *P. m. dilutus* (Richmond, 1896; NW China SW Mongolia), and *P. m. saturatus* (Stejneger 1885; Summer-Smith 1995; Del Hoyo et al. 2018; South Korea, Taiwan, Japan, Sakhalin). Genetic research on complete mitochondrial genome (mitogenome) sequence of the *P. m. saturatus* was conducted at Anhui, China (Yang et al. 2016), but it has not been done in the Republic of Korea. In this study, we determined the whole mitogenome sequence of the *P. m. saturatus* and conducted phylogenetic analysis with related species. This study will help determining the phylogenetic position and evolution of the genome of this species.

To get the biological sample, a male individual *P. m. saturatus* (Ring color: blue green & sky) was captured from the Agricultural Practical Training Center (N 35°10'31.02", E 126°53'56.33", altitude: 40 m) at Chonnam National University, using mist net on 26 February 2020. We collected blood sample from capillary vessel by using Microsyringes, and stored the sample in EDTA vial (Voucher no. CNU2020-TSB140) at −20 °C, Research Center of Ecomimetics in Chonnam National University. The total genomic DNA was extracted by using the DNeasy Blood and Tissue kit (Qiagen, Valencia, CA) according to the manufacturer’s protocol, and the extracted DNA was stored at the −20 °C freezer. In this study, we sequenced the complete mitogenome of the *P. m. saturatus* by PCR-based approach (PRINZA 692774). We determined the complete mitogenome sequence using the direct sequencing (1000-bp length in each read) generated from Applied Biosystems 3730XL DNA Analyzer (Bionics Co., Seoul, Republic of Korea). Sequences arranged using *de novo* assembly and annotation by comparing *P. montanus* mitogenome (Accession no. JX486030) and *P. m. saturatus* mitogenome (Accession no. KM577704) in GenBank, and analysis mitogenome structure using Geneious prime 2021.0.3 (www.geneious.com). Phylogenetic analysis was constructed using maximum-likelihood method (Rzhetsky and Nei 1992) using MEGA X software with 100 replicates bootstrap method (Kumar et al. 2018).

**Figure 1.**
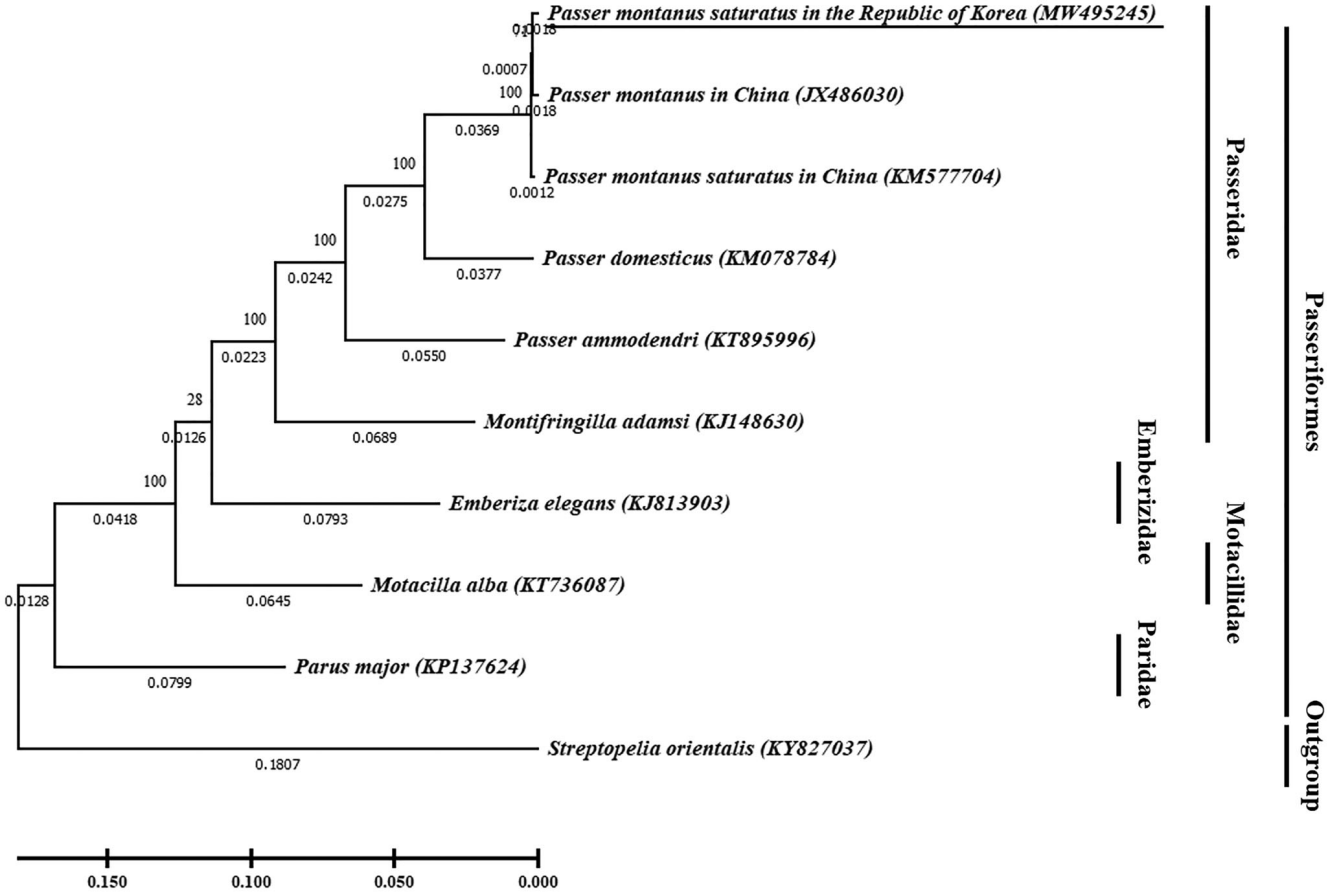
Phylogenetic tree of *Passer montanus saturatus* in the Republic of Korea (underlined; MW495245) and other related species based on mitochondrial (mt) genome full sequence data. *Streptopelia orientalis* was used as an outgroup. In the phylogenetic tree, evolutionary distances were expressed as branch length. The evolutionary distances computed using the maximum likelihood method, and were in the units of the number of base substitutions per site. Bootstrap method used 100 replicates to know statistical support. The phylogenetic analysis was performed using MEGA X (Kumar et al. 2018).

The complete mitogenome of *P. m. saturatus* was 16,904 bp in length deposited in GenBank (Accession no. MW495245), and contains 13 protein-coding genes (PCGs), 22 transfer RNA (tRNA) genes, 2 ribosomal RNA genes (rRNA), and a putative long non-coding control region (NCR). The order and orientation are identical with that of the standard avian gene order, and other *P. montanus*’s mitogenome sequence (Gibb et al. 2006; Yang et al. 2016). The nucleotide composition of the *P. m. saturatus* in the Republic of Korea (A = 30.0%, T = 22.5%, G = 15.5%, C = 31.9%) was similar to that of *P. montanus* in China (JX486030.1; A = 30.0%, T = 22.5%, G = 15.5%, C = 31.9%), and *P. m. saturatus* in China (KM577704.1; A = 30.0%, T = 22.5%, G = 15.5%, C = 31.9%). Comparison of sequences between *P. m. saturatus* in the Republic of Korea and *P. montanus.* in China indicated a 99.62% sequence identity, and comparison with *P. m. saturatus* in China was 99.61% identity (Figure 1).

For phylogenetic analysis to investigate genetic relationship of *P. montanus saturatus* in Korea, the evolutionary history was inferred by using the maximum likelihood method with Tamura-Nei model (Tamura and Nei 1993). The full mitogenome sequences of nine Passeriformes were extracted from Genbank, with *Streptopelia orientalis* used as an outgroup. The phylogenetic tree showed that the close relationship between *P. m. saturatus* in the Republic of Korea and *P. montanus* in China (JX486030) than *P. m. saturatus* in China (KM577704). The phylogenetic relationship showed that the genetic distance increased as it progressed to the Passeridae, Emberizidae, Motacillidae, and Paridae in the Passeriformes. These data provide genetic information for *P. m. saturatus* resided in the Republic of Korea, for further studies about geographical species distribution of the Eurasian Tree Sparrow.

## Data Availability

GenBank accession number and BioProject Accession number from the complete mitochondrial genome of *Passer montatus* subsp. *saturatus* (GenBank Accession no. MW495245, BioProject Accession no. PRJNA692774) has been registered with the NCBI database. A specimen was deposited at Research Center of Ecomimetics in CNU under the sample number CNU2020-TSB140 (E-mail address of the person in charge: schol2002@jnu.ac.kr).
